# WITHDRAWAL: Congenital Granular Cell Epulis in a Newborn: Diagnostic and Surgical Challenges in a Resource‐limited Setting—a Case Report

**DOI:** 10.1002/ccr3.73286

**Published:** 2026-08-06

**WITHDRAWAL**: A. I. Hassan , A. A. Sheikh , B. M. Kulle , E. M. Jama , F. A. Mohamud , and A. M. Bashir , “Congenital Granular Cell Epulis in a Newborn: Diagnostic and Surgical Challenges in a Resource‐limited Setting—a Case Report,” Clinical Case Reports 14, no. 5 (2026): e72524, 10.1002/ccr3.72524.42051968 PMC13111420

The above article, published online on 26 April 2026 in Wiley Online Library (wileyonlinelibrary.com), has been withdrawn by agreement between the authors; the journal Editor‐in‐Chief, Dr. Roberto Berebichez‐Fridman; and John Wiley & Sons Ltd. The withdrawal has been agreed to because informed consent for publication of identifying details was not properly secured from the legal guardians of the patient featured in the case report.

